# Synergistic impact of arbutin and kaempferol-7-*O*-α-l-rhamnopyranoside from *Nephelium lappaceum* L. on whitening efficacy and stability of cosmetic formulations

**DOI:** 10.1038/s41598-023-49351-3

**Published:** 2023-12-12

**Authors:** Ittipon Siridechakorn, Junsuda Pimpa, Siwattra Choodej, Nattaya Ngamrojanavanich, Khanitha Pudhom

**Affiliations:** 1 Department of Chemistry, Faculty of Science, Chulalongkorn University, Bangkok, 10330 Thailand; 2 Department of Chemistry, Faculty of Science, King Mongkut’s University of Technology, Thonburi, 10140 Thailand

**Keywords:** Health care, Chemistry

## Abstract

Four flavonoid glycosides, namely quercetin-3-*O*-rhamnoside (**1**), kaempferol-3-*O*-β-d-glucopyranosyl (**2**), kaempferol-7-*O*-α-l-rhamnopyranoside (**3**), and kaempferol-3-*O*-β-d-glucopyranosyl-7-*O*-α-l-rhamnopyranoside (**4**), from *Nephelium lappaceum* L. seeds were evaluated for their efficacy against melanin inhibition in B16F10 melanoma cells and tyrosinase inhibition. Among them, kaempferol-7-*O*-α-l-rhamnopyranoside (**3**) displayed the highest potency in both activities without any significant cytotoxicity. The combination of compound **3** and arbutin in specific proportions demonstrated a synergistic effect (CI < 1) in inhibiting melanin production in B16F10 cells and tyrosinase inhibition. Additionally, a cosmetic formulation containing compound **3** and arbutin as active ingredients exhibited favorable stability under accelerated storage conditions. Quantitative analysis indicated that compound **3** and arbutin levels in the formulation were above 90% after one month of storage. Determination of the formulation's shelf life using the Q10 method, estimating it to be around 5.2 months from the date of manufacture. The synergy between arbutin and kaempferol-7-O-α-l-rhamnopyranoside (**3**) extracted from *N. lappaceum* substantially enhances both the whitening effectiveness and the stability of cosmetic formulations.

## Introduction

Melanin, a pigment responsible for shielding the skin against ultraviolet (UV) damage and regulating skin pigmentation, plays a pivotal role in maintaining skin integrity^1^. However, excessive melanin production and accumulation lead to various skin pigmentation disorders, including melasma, freckles, and dark spots^2^. Hyperpigmentation profoundly affects an individual's physical appearance and self-assurance in social interactions. Presently, the treatment of hyperpigmentation entails the utilization of whitening agents, chemical peels, and laser therapy^3^. Among these modalities, common topical treatment with effective skin lightening/whitening agents is still necessary due to the resistant and relapsing nature of hyperpigmentation^4^. Indeed, various commercially available lightening agents, such as kojic acid, hydroquinone, retinoids, have proven to be effective for topical management; however, most of them caused undesirable side effects, including possible carcinogenicity and dermatitis^4–6^. Moreover, hydroquinone and kojic acid are prohibited in most countries. Consequently, the need to discover safer and more effective melanogenic inhibitors persists.

Thailand is renowned for its agricultural prominence, and *Nephelium lappaceum* L. (known as rambutan), is one of the economically significant crops in Thailand. In addition to commercial fresh fruits, rambutan is industrially processed into can, juice, jam, jelly, and spread^7^. This processing produces a large amount of seed waste, a yearly average 1900 tons discarded^8^. There is growing concern about agricultural wastes that contain bioactive compounds. Previous phytochemical investigations demonstrated various phenolic compounds were found in rambutan seed, some of which exhibited antioxidant, antimicrobial, and nitric oxide inhibition^9–11^.

To enhance the economic value of such kind of waste with a growing preference for natural products within the cosmetic industry, rambutan roots-derived phenolics and their melanin and tyrosinase inhibition have been studied. Furthermore, we aimed to explore the synergistic effect of the combination of the potential compounds and melanin-inhibiting whitening agents and/or tyrosinase inhibitors, due to research on synergistic interaction of two or more compounds attracted great interest in recent years^12^. In addition to greater impact on biological activity, synergy can also influence the physicochemical properties of compounds or mixtures, including solubility and absorption efficiency^13^. Finally, the chemical stability of both these substances will be examined within cosmetic formulations.

## Results and discussion

### Anti-melanin activity and cytotoxicity of compounds 1–4

**Figure 1:**
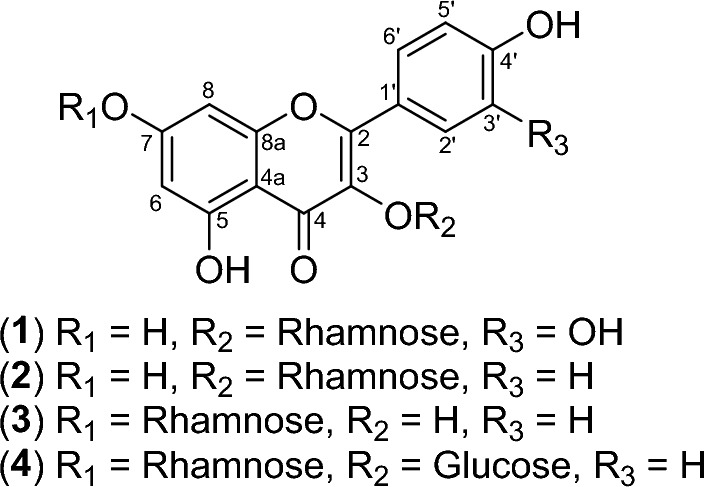
Structures of compounds **1–4** isolated from *N. lappaceum* L. seed extract.

**Figure 2:**
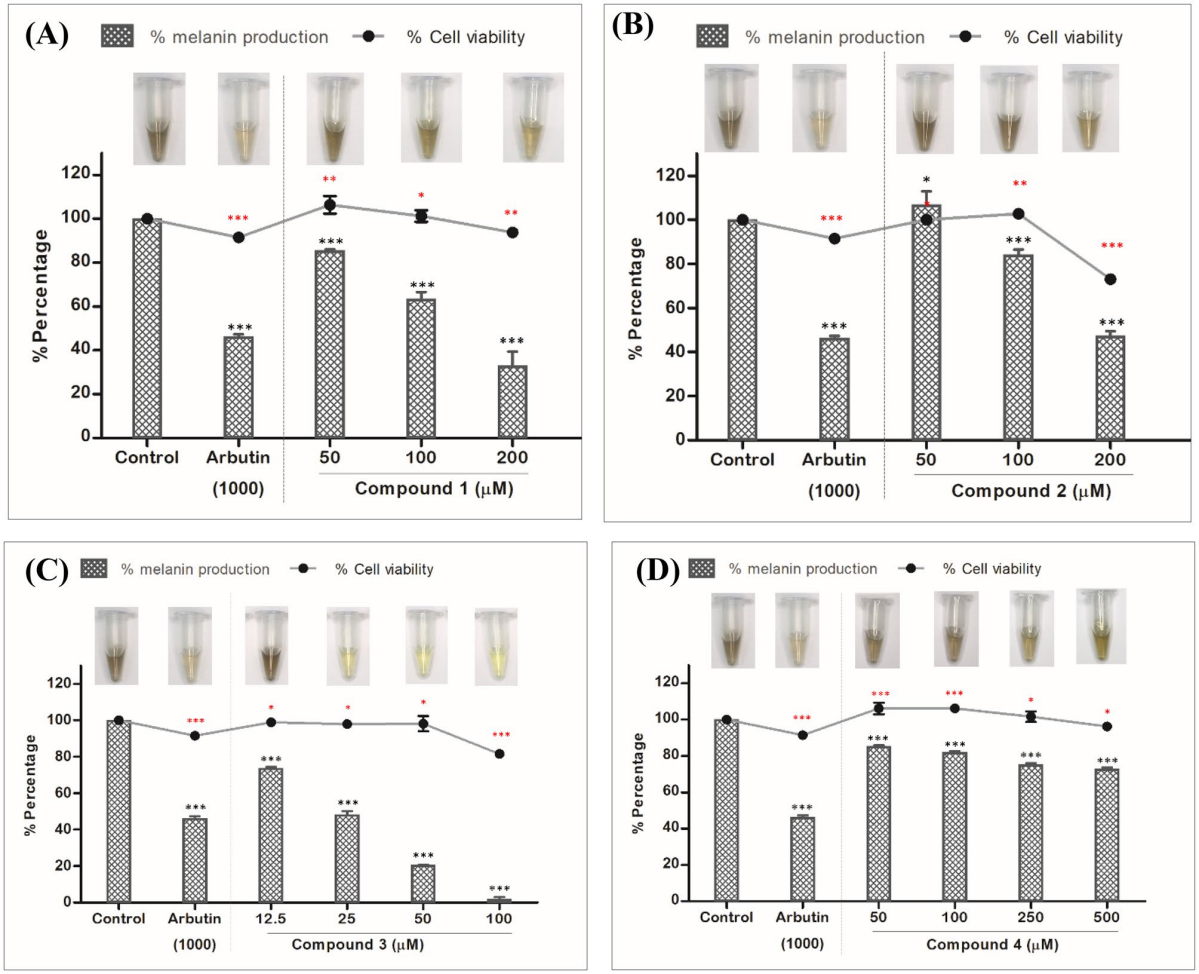
Effect of various concentrations of compounds **1**–**4** (**A**–**D**) on the melanin content in B16F10 melanoma cells. Following exposure, cell viability was then measured by MTT assay. The percentages are plotted as the mean ± SD of triplicate. *P < 0.05, **P < 0.01 and ***P < 0.001 compared with untreated control.

To assess the effectiveness of phytochemicals isolated from the *N. lappaceum* L. seed extract in inhibiting melanin production, a B16F10 cell-based anti-melanogenic assay was conducted on four specifically chosen compounds (**1**–**4**) (Fig. 1). As a positive control, arbutin, a widely recognized tyrosinase inhibitor frequently employed as a cosmetic whitening agent, was utilized^14^. Among them, compound **3** exhibited the most potent anti-melanogenic activity in a concentration-dependent manner, while no significant toxicity was observed (Fig. 2C). It provided an IC_50_ value of 23.0 ± 1.3 µM, indicating compound **3** was about 35-fold more potent than positive control arbutin (IC_50_ = 807.6 ± 2.9 µM) (Table 1). The remaining three compounds **1**–**2** and **4** could also suppress melanin production in B16F10 cells in a dose-dependent manner, with IC_50_ values of 134.8 ± 2.1, 190.5 ± 2.2, and > 200 µM, respectively (Fig. 2A,B,D). From these data, some structure–activity relationships could be seen. The position of the rhamnose moiety might play an important role in melanin inhibition and cytotoxicity. Attachment of rhamnose at the C-7 position to kaempferol core skeleton as in **3** exerted a remarkably potent melanin reduction, whereas the C-3 existence of rhamnose in **2** caused much decrease in anti-melanogenic activity but increase in cellular toxicity. In addition, it was implied that the presence of a glucosyl moiety at C-3 position as in **4** made the compound loss of both melanin suppression and toxicity. Surprisingly, Tang et al. previously reported that kaempferol itself did induce melanin production and melanocyte growth^15^. The results indicated that the existence of the sugar moiety is required for melanin inhibition of the kaempferol core skeleton.

**Table 1:** Anti-melanogenic activity of compounds **1–4** and arbutin on B16F10 melanoma cells.

Compounds	IC_50_ (µM)	Cytotoxicity (µM)
**1**	134.8 ± 2.1	> 200
**2**	190.5 ± 2.2	200
**3**	23.0 ± 1.3	> 1000
**4**	> 200	> 1000
Arbutin	807.6 ± 2.9	> 1000

Data are expressed as mean ± SD (n = 3).

### Anti-tyrosinase activity of compound 3

Tyrosinase plays a pivotal role as an essential enzyme, exhibiting multifarious catalytic functionalities within the melanogenesis process^16^. Hence, inhibiting tyrosinase represents a crucial strategy in addressing disorders associated with pigmentation. In this study, the inhibitory activity of the compound on tyrosinase was evaluated through the employment of a mushroom tyrosinase assay. Based on the anti-melanin activity, compound **3** was chosen for subsequent investigation owing to its pronounced melanin inhibition activity, in addition to the absence of discernible cytotoxicity even after 72 h of treatment with concentrations as high as 1000 µM. Most notably, its anti-melanogenic activity exhibited a 35-fold superiority to that of arbutin. Compound **3** exhibited strong anti-tyrosinase activity, with an IC_50_ value of 25.3 ± 0.1 µM, comparable to kojic acid (positive control) and much potent than arbutin (active ingredient in commercial skincare products) (Table 2). This heightened activity might be attributed to the hydroxyl groups present in kaempferol, which facilitate binding to the enzymatic site of tyrosinase. Consequently, this binding impedes the catalytic oxidation of L-DOPA to L-DOPA quinone via catecholate activity^17^.

**Table 2:** Anti-tyrosinase activity of compound **3**, arbutin and kojic acid.

Sample	IC_50_ (µM)
Compound **3**	25.3 ± 0.1
Arbutin*	1166 ± 0.04
Kojic acid**	39.0 ± 0.02

*Active ingredient in commercial skincare products.

**Positive control.

### Synergistic Impact of arbutin and kaempferol-7-*O*-α-l-rhamnopyranoside (3)

**Figure 3:**
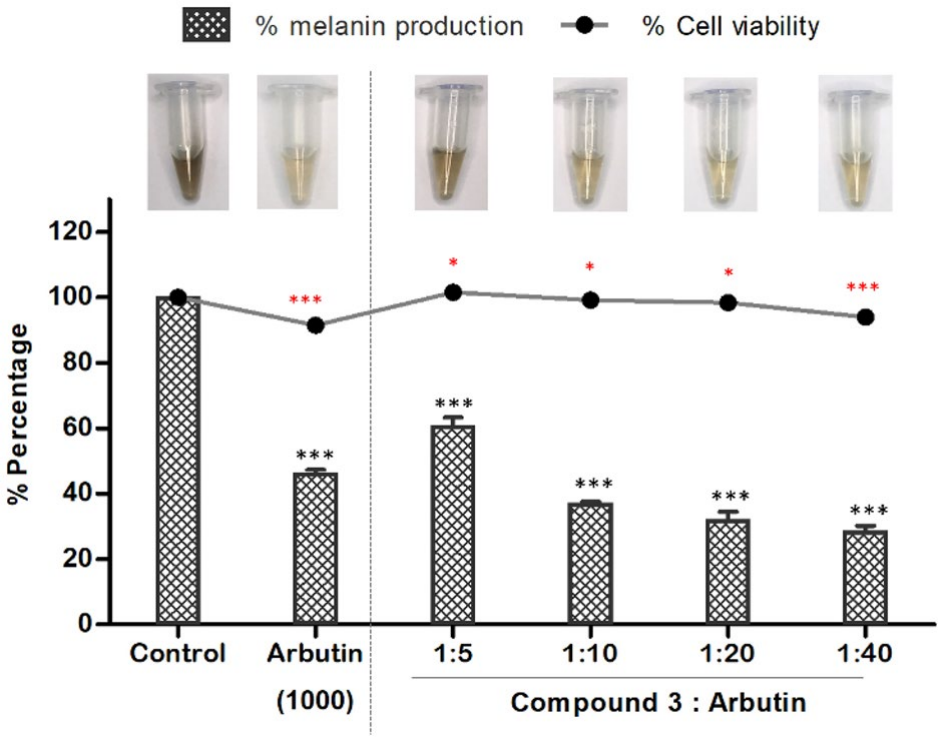
Effect of the mixtures of compound **3** and arbutin with various ratio on the melanin content in B16F10 melanoma cells. Following exposure, cell viability was then measured by MTT assay. The percentages are plotted as the mean ± SD of triplicate. *P < 0.05, **P < 0.01 and ***P < 0.001 compared with untreated control.

Recent research findings have revealed the application of arbutin, a hydroquinone compound, and kojic acid as potent tyrosinase inhibitors within the realm of cosmetic formulations. Nevertheless, the usage of hydroquinone has been associated with undesirable consequences, including irritative responses, dermatitis, and the impairment of melanin. Furthermore, arbutin derivatives undergo catalysis, leading to the production of benzene metabolites that may possess inherent hematotoxicity^18,19^. Furthermore, when kojic acid is utilized at concentrations exceeding 1%, it has the potential to elicit skin irritations, contact dermatitis, and increased vulnerability to sunburn, particularly in individuals possessing sensitive skin conditions^20^. The synergistic effect is a phenomenon that arises when two or more substances collaborate to yield an aggregated impact surpassing the cumulative effect of their individual properties. Moreover, the synergistic effects may extend to influence the physicochemical characteristics of compounds or mixtures, such as solubility^12,13^. Due to lower side effects, arbutin, a natural β-d-glucopyranoside of hydroquinone, has gained widespread employment as a depigmentation agent within the cosmetic industry. In current study, compound **3** was selected to be synergistically combined with arbutin to investigate their collective impact on suppressing tyrosinase activity and inhibiting melanin production in B16F10 cells. Various combinations of compound **3** and arbutin were prepared, utilizing ratios of 1:5, 1:10, 1:20, and 1:40 to reveal a notable reduction in melanin content within B16F10 cells (Fig. 3). Treatment with compound **3** and arbutin mixtures resulted in reductions of 39.4%, 63.1%, 68.0%, and 71.5% in melanin content, respectively. These sample mixtures were further subjected to evaluation of their anti-tyrosinase activity, which manifested as a reduction in inhibitory percentages of 23.5%, 27.3%, 37.0%, and 49.6%, respectively (Table 3). The calculation of combination index (CI) values revealed that the mixtures comprising compound **3** and arbutin exhibited synergistic effects, as evidenced by CI values below 1.0 (Table 3). Notably, treatment with a 1:40 mixture of compound **3** and arbutin demonstrated the most potent inhibitory effects on melanin production in B16F10 cells, resulting in a remarkable reduction of up to 75.6%. These findings indicated that the combined mixture of compounds exhibits enhanced efficacy as melanin inhibitors compared to their individual extracts. Additionally, Table 3 provides a summary of the percentage inhibition of tyrosinase activity and the corresponding CI values of the mixtures. The results indicated that the mixtures of compound **3** and arbutin also displayed a synergistic effect, as indicated by CI values below 1.0.

**Table 3:** Anti-melanin and anti-tyrosinase activities of the mixtures of compound **3** and arbutin.

Concentration (µM)		Ratio	Anti-melanin activity		Anti-tyrosinase activity	
Compound **3**	Arbutin		% Inhibition	CI value	% Inhibition	CI value
12.5	62.5	1:5	39.4 ± 2.2	0.78	23.5 ± 3.4	0.92
12.5	125	1:10	63.1 ± 0.5		27.3 ± 4.3	
12.5	250	1:20	68.0 ± 2.0		37.0 ± 2.6	
12.5	500	1:40	71.5 ± 1.3		49.6 ± 1.6	

### Stability of cosmetic formulations

#### Physical stability

**Figure 4:**
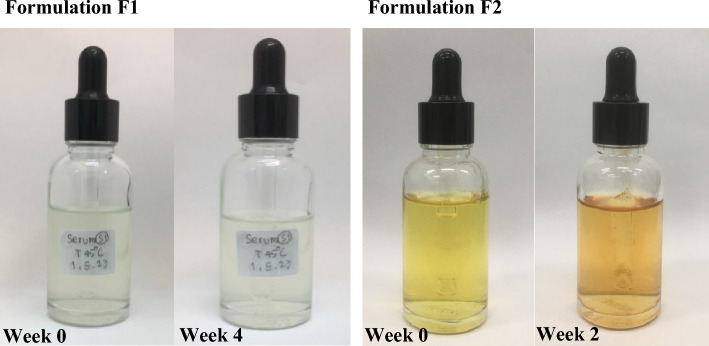
Color of the formulation **F1** and **F2** in the accelerated storage period.

The physical attributes of formulation **F1** were examined, revealing no discernible alterations in color, odor, or texture. Moreover, **F1** demonstrated homogeneity, maintaining its uniformity without any indications of phase separation during centrifugation (Table 4). Additionally, over the duration of the accelerated storage period, **F1** displayed consistent pH values, with no significant deviations detected. In contrast, it was observed that formulation **F2** experienced a color change after two weeks of the study (Fig. 4). The observed phenomenon can be attributed to the utilization of triethanolamine as a pH-adjusting base in formulation **F2**, aimed at achieving an optimal pH range for the product. Subsequently, alkaline hydrolysis^21^ took place specifically with compound **3**, leading to a discernible alteration in the product color right from the initiation of the formulation process. Furthermore, during the storage period under accelerated conditions, the color change became increasingly conspicuous and evident.

**Table 4:** Physical stability test of formulation **F1** and **F2**.

Organoleptic test		Formulation F1	Formulation F2
Color change		Not change	Change
Odor change		Not change	Not change
Phase separation		Not change	Not change
pH	Weeks		
	0	5.88 ± 0.01	6.85 ± 0.00
	1	5.88 ± 0.01	7.41 ± 0.07
	2	5.86 ± 0.01	7.50 ± 0.01
	3	5.85 ± 0.02	7.44 ± 0.05
	4	5.84 ± 0.01	7.44 ± 0.05

#### Chemical stability

**Figure 5:**
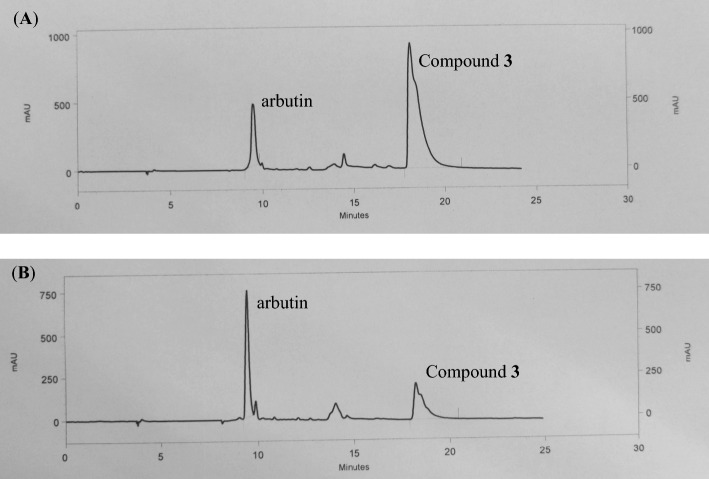
HPLC chromatogram of standard compound **3** and arbutin (**A**), and formulation **F1** (**B**).

**Figure 6:**
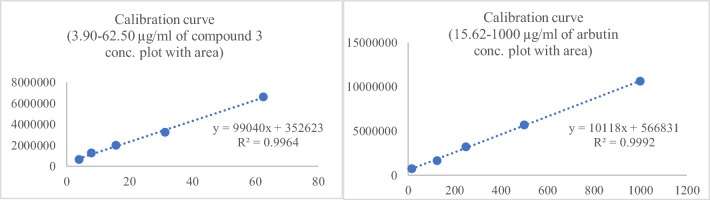
Calibration curve of compound **3** and arbutin.

Formulation **F1** was employed for assessing the chemical stability, as it exhibited consistent physical stability under accelerated conditions without any observable changes. Performing the HPLC analysis using a single peak chromatogram of standard compounds, compound **3** and arbutin to generate the calibration curve (Fig. 5). Calibration curves of compound **3** and arbutin were performed at different concentrations (compound **3**: 3.90, 7.81, 15.62, 31.25, 62.50 µg/mL, arbutin: 15.62, 125, 250, 500, 1000 µg/mL) (Fig. 6). The amount of each compound** 3** and arbutin was calculated based on a linear equation: Y = 99040X + 352,623, R^2^ = 0.9964 and Y = 10118X + 566,831, R^2^ = 0.9992, respectively (Fig. 6). Each calibration point was conducted in triplicate. The formulation **F1** contained residual quantities of compound **3** and arbutin, with percentages of 96.00% and 97.44%, respectively (Table 5). Through the calculation of the remaining percentage of both active ingredients in the facial serum, it was established that the quantities of these active ingredients in the formulation remained above 90%^22^ after 1 month. Additionally, by employing the experimental results and the Q10 method for estimation, it was deduced that the serum possessed a maximum predicted shelf life of 5.2 months, during which no discernible alterations in its chemical and physical properties were observed^23,24^.

**Table 5:** Compound **3** and arbutin contents in formulation** F1**.

Compounds	LOD (%w/w)	LOQ (%w/w)	Formulation **F1** (%w/w)		%Remaining
			Day 1	Day 30	
Compound **3**	0.0016	0.0048	0.0050 ± 0.0005	0.0048 ± 0.0003	96.00%
Arbutin	0.0124	0.0375	0.1991 ± 0.0004	0.1940 ± 0.0057	97.44%

*LOD* limit of detection, *LOQ* limit of quantification.

Calculation for predicting the shelf life of a formulation using the Q10 method.

$${{\text{t}}}_{30}=\frac{{{\text{t}}}_{30}({{\text{T}}}_{1})}{{{Q}_{10}}^{(\frac{\Delta {\text{t}}}{10})}} = \frac{1}{{3}^{(\frac{-15}{10})}}= 5.2\mathrm{ months}$$

where, T_90_(T_1_) = Shelf life at 45 °C (T_1_); T_90_(T_2_) = Estimated shelf life at 30 °C (T_2_); ∆T = Temperature difference between T2 and T1 (30–45 = − 15); Q_10_ = Ratio of the rate of reaction with a temperature difference of 10 °C (3 is constant).

## Conclusion

Four flavonoid glycosides isolated from *N. lappaceum* L. seeds were assessed for their anti-melanogenic activity in B16F10 melanoma cells. Among them, kaempferol-7-*O*-α-l-rhamnopyranoside (**3**) exhibited the most potent activity without significant cytotoxicity. Moreover, it displayed remarkable anti-tyrosinase activity comparable to that of kojic acid, making it a promising candidate as an active ingredient in skin whitening products. Our study also demonstrated a synergistic compatibility effect when combining compound **3** and arbutin in specific proportions, assessed using the CI value to evaluate synergism. The results revealed a synergistic effect (CI < 1) between compound **3** and arbutin in inhibiting melanin production in B16F10 cells and tyrosinase inhibition using L-tyrosine as a substrate. Consequently, we selected compound **3** and arbutin as active ingredients for inclusion in cosmetic formulations. Furthermore, formulation **F1** demonstrated excellent stability under all accelerated storage conditions, and the quantitative analysis confirmed that the levels of compound **3** and arbutin in the formulation remained above 90% after one month of storage. To determine the shelf life of the formulation, we utilized the Q10 method, which estimates a shelf life of approximately 5.2 months from the date of manufacture. In conclusion, the collaborative effect of arbutin and kaempferol-7-O-α-l-rhamnopyranoside (**3**) derived from *N. lappaceum* significantly enhances both the whitening efficacy and the stability of cosmetic formulations.

## Materials and methods

### Materials

Quercetin-3-*O*-rhamnoside (**1**), kaempferol-3-*O*-β-d-glucopyranosyl (**2**), kaempferol-7-*O*-α-l-rhamnopyranoside (**3**), and kaempferol-3-*O*-β-d-glucopyranosyl-7-*O*-α-l-rhamnopyranoside (**4**) were isolated from the air-dried powdered seeds of *N. lappaceum* L. with several chromatographic techniques as our previous report^25^. The *N. lappaceum* (Sapindaceae) seeds were gathered in Amphoe Laem Sing, Chanthaburi Province, Thailand. All methods were carried out in accordance with relevant guidelines. Arbutin, 3-(4,5-dimethylthiazol-2-yl)-2,5-diphenyltetrazolium bromide (MTT), kojic acid and Tween 20 were purchased from Tokyo Chemical Industry (Japan). L-Tyrosine and mushroom tyrosinase were purchased from Sigma-Aldrich (USA). Dulbecco’s Modified Eagle Medium (DMEM), penicillin/streptomycin, trypan blue solution, trypsin 0.25 (1×) solution were purchased from Hyclone (Austria). The chemicals for cosmetic formulation, which comprised glycerin, propylene glycol, xanthan gum, ActiveProtecTMOX (consisting of Sodium Diethylenetriamine Pentamethylene Phosphonate, Disodium EDTA, Sodium Metabisulfite, and Sodium Gluceptate), arbutin, phenoxyethanol, hydroxyethyl cellulose, Acrylates/C10-30 Alkyl Acrylate Crosspolymer, and triethanolamine, were procured from Chanjao Longevity Co., Ltd. (Thailand).

### Methods

#### Cell culture

Murine melanoma B16F10 cells (obtained from DS Pharma Biomedical, Japan) were cultivated in Dulbecco's Modified Eagle Medium (DMEM), a liquid medium supplemented with 10% fetal bovine serum (FBS) and 1% penicillin/streptomycin. The cells were incubated at a temperature of 37 °C within a controlled humidified environment containing 5% CO_2_. To facilitate subculture, the cells were detached from the culture flask using trypsin solution. Subsequently, the cells were subjected to staining with trypan blue solution and quantified using a hemocytometer for further analysis. The final concentration of DMSO was 0.1% for all experiments.

#### Anti-melanogenic assay

Murine melanoma B16F10 cells were gently rinsed with phosphate-buffered saline (PBS) and detached from the cell tissue culture dish using trypsin. The cells were subsequently seeded in 24-well culture plates at a density of 5 × 10^4^ cells per well and allowed to incubate for 24 h before being subjected to treatment with the experimental samples. Arbutin was used as a positive control. Following the treatment, the cells were further incubated for an additional 72 h, and the absorbance was measured at 510 nm using a microplate reader. Melanin production was expressed as a percentage relative to the control cells treated with DMSO. All experiments were conducted in triplicate. The IC_50_ value was calculated. The percentage of total melanin content was determined using the following equation:

$$\mathrm{\% Total\, melanin\, content }= \frac{{\text{Abs}}\left(\text{treated cells}\right)-{\text{Abs}}\left(\text{blank of treated cells}\right)}{{\text{Abs}}\left({\text{control}}\right)-\text{ Abs}\left(\text{blank of control}\right)}\times 100$$

#### Cytotoxicity assay

Cytotoxicity was assessed through the utilization of the MTT assay. The cell culture was initiated in 24-well plates, with a seeding density of 5 × 10^4^ cells per well. After treatment with samples for 72 h, 10 µL of MTT solution (5 mg/mL in PBS) were added to each well and incubated for additional 3 h. Then, supernatant was removed and DMSO (250 µL) was added to dissolve the formed formazan crystals. The absorbance was measured at 570 nm using a microplate reader. Cells treated solely with DMSO served as the control. All experiments were conducted in triplicate. The percentage of cell viability was determined using the following calculation:

$$\mathrm{\% Cell\, viability }= \frac{{\text{Abs}}\left(\text{treated cells}\right)-{\text{Abs}}\left(\text{blank of treated cells}\right)}{{\text{Abs}}\left({\text{control}}\right)- \, {\text{Abs}}\left(\text{blank of control}\right) \, }\times 100$$

#### Anti-tyrosinase assay

The assessment of tyrosinase inhibitory activity was conducted following the method outlined by Saewan et al., with slight modifications^26^. l-tyrosine served as a substrate and kojic acid was utilized as a positive control. The compounds with various concentrations (50 μL) were added to the 96-well plate, followed by tyrosinase solution (50 μL, 250 units/mL in phosphate buffer, pH 6.8). After the first incubation at 35 °C for 10 min, 50 μL of substrates (2.5 mM l-tyrosine) were added, followed by second incubation at 35 °C for 20 min. The absorbance was measured at 492 nm using a microplate reader. All experiments were replicated in triplicate. The percentage inhibition of tyrosinase was determined using the following equation:

$$\mathrm{\% Tyrosinase\, inhibition }= \frac{{\text{Abs}}\left({\text{control}}\right) -\text{ Abs}\left({\text{sample}}\right)}{{\text{Abs}}\left({\text{control}}\right) \, -\text{ Abs}\left(\text{sample of control}\right) \, }\times 100$$

#### Cosmetic formulation and accelerated stability test

The development of cosmetic formulations has resulted in the creation of two distinct formulations, outlined as follows. Formulation **F1** consists of the following ingredients: compound **3** (0.005% w/w), glycerin (3.00% w/w), propylene glycol (3.00% w/w), xanthan gum (0.5% w/w), ActiveProtecTMOX (Sodium Diethylenetriamine Pentamethylene Phosphonate, Disodium EDTA, Sodium Metabisulfite, Sodium Gluceptate) (0.60% w/w), arbutin (0.20% w/w), phenoxyethanol (0.5% w/w), and water (92.195% w/w). Formulation **F2** consists of the following ingredients: compound **3** (0.005% w/w), glycerin (2.00% w/w), propylene glycol (1.00% w/w), hydroxyethyl cellulose (0.5% w/w), Acrylates/C10-30 Alkyl Acrylate Crosspolymer (0.10% w/w), triethanolamine (0.05% w/w), phenoxyethanol (0.5% w/w) and water (95.845% w/w). An accelerated stability test was performed by placing the formulation in a 45 °C chamber for 30 days. The physical and chemical stability of the test products were assessed through organoleptic and HPLC analyses.

#### Determination of kaempferol-7-O-α-l-rhamnopyranoside (3) and arbutin in cosmetic formulation by HPLC

The stock solution was prepared by dissolving 1 mg of the standard compounds in 1 mL of methanol, resulting in a concentration of the stock solutions at 1000 ppm. Subsequently, serial dilutions were conducted using methanol to establish a standard curve. The concentrations varied from 3.9 to 62.5 ppm for kaempferol 7-*O*-α-l-rhamnopyranoside (**3**) and from 15.6 to 1000 ppm for arbutin. For the analysis of the sample cosmetic formulations, precisely 200 mg was weighed and placed into a centrifuge tube, then dissolved in 1 mL of methanol. The resulting mixture was vortexed for 1 min, followed by sonication at 25 °C for 15 min and subsequent freezing at − 80 °C for 10 min. The mixture was then subjected to centrifugation at 3000 rpm for 5 min. The sample solution was filtered through a nylon syringe filter with a pore size of 0.45 µm, and 20 μL of the filtrate was injected into a reverse-phase Vertical C18 column (150 mm × 4.6 mm, 5 μm) equipped with a Thermo Scientific UV6000LP detector and Thermo Scientific P200 pump. The ChemQuest version 5.0 software controlled the system. The mobile phase, consisting of water (A) and acetonitrile (B), followed a linear gradient program of 0–100% B over 30 min, with a flow rate of 0.6 mL/min. The UV detector was set at 280 nm, and the injection volume was 20 μL.

#### Statistical analysis

The 50% inhibition concentration (IC_50_) value is determined with GraphPad prism software, version 5. Data were presented as mean ± standard deviation (SD) from three independent experiments. Values were evaluated by two-way analysis of variance (ANOVA), followed by Bonferroni correction for multiple comparisons using GraphPad Prism version 5. *p < 0.05, **p < 0.01 and ***p < 0.001 indicate statistical significance (compared with untreated control). The values of combination index (CI) were calculated by following equation:

$${\text{CI}} \, \text{=} \, \frac{{\text{IC}}_{\text{50amix}}}{{\text{IC}}_{\text{50a}}}\text{+}\frac{{\text{IC}}_{\text{50bmix}}}{{\text{IC}}_{\text{50b}}}$$

where IC_50a_ and IC_50b_ are the IC_50_ value of compound a and compound b, and IC_50amix_ and IC_50bmix_ are the concentration of compound a and compound b in the mixture that cause 50% inhibition. The CI value < 1 indicates synergism, CI = 1 indicates an additive effect, and CI > 1 indicates antagonism^15^.

## Data Availability

All data and materials are the result of research and are available from the corresponding author upon reasonable request and are appropriately cited in the manuscript.
